# Effect of β-hydroxybutyrate on behavioral alterations, molecular and morphological changes in CNS of multiple sclerosis mouse model

**DOI:** 10.3389/fnagi.2022.1075161

**Published:** 2022-12-01

**Authors:** Wei Sun, Min Wen, Min Liu, Qingpeng Wang, Quiqin Liu, Lanjie Li, Hans-Christian Siebert, Gabriele Loers, Ruiyan Zhang, Ning Zhang

**Affiliations:** ^1^Institute of Biopharmaceutical Research, Liaocheng University, Liaocheng, Shandong, China; ^2^Shandong Donkey Industry, Technology Collaborative Innovation Center, Liaocheng University, Liaocheng, China; ^3^Schauenburgerstr, RI-B-NT - Research Institute of Bioinformatics and Nanotechnology, Kiel University, Kiel, Germany; ^4^Center for Molecular Neurobiology Hamburg, University Medical Center Hamburg-Eppendorf, University of Hamburg, Hamburg, Germany; ^5^Laboratory of Food Science and Technology, Jiangnan University, Wuxi, China

**Keywords:** multiple sclerosis, **β**-hydroxybutyrate, demyelination, TRPA1, PARP

## Abstract

Multiple sclerosis (MS) is a chronic inflammatory and degenerative disease of central nervous system (CNS). Aging is the most significant risk factor for the progression of MS. Dietary modulation (such as ketogenic diet) and caloric restriction, can increase ketone bodies, especially β-hydroxybutyrate (BHB). Increased BHB has been reported to prevent or improve age-related disease. The present studies were performed to understand the therapeutic effect and potential mechanisms of exogenous BHB in cuprizone (CPZ)-induced demyelinating model. In this study, a continuous 35 days CPZ mouse model with or without BHB was established. The changes of behavior function, pathological hallmarks of CPZ, and intracellular signal pathways in mice were detected by Open feld test, Morris water maze, RT-PCR, immuno-histochemistry, and western blot. The results showed that BHB treatment improved behavioral performance, prevented myelin loss, decreased the activation of astrocyte as well as microglia, and up-regulated the neurotrophin brain-derived neurotrophic factor in both the corpus callosum and hippocampus. Meanwhile, BHB treatment increased the number of MCT1^+^ cells and APC^+^ oligodendrocytes. Furthermore, the treatment decreased the expression of HDAC3, PARP1, AIF and TRPA1 which is related to oligodendrocyte (OL) apoptosis in the corpus callosum, accompanied by increased expression of TrkB. This leads to an increased density of doublecortin (DCX)^+^ neuronal precursor cells and mature NeuN^+^ neuronal cells in the hippocampus. As a result, BHB treatment effectively promotes the generation of PDGF-Ra^+^ (oligodendrocyte precursor cells, OPCs), Sox2^+^ cells and GFAP^+^ (astrocytes), and decreased the production of GFAP^+^ TRAP1^+^ cells, and Oligo2^+^ TRAP1^+^ cells in the corpus callosum of mouse brain. Thus, our results demonstrate that BHB treatment efficiently supports OPC differentiation and decreases the OLs apoptosis in CPZ-intoxicated mice, partly by down-regulating the expression of TRPA1 and PARP, which is associated with the inhibition of the p38-MAPK/JNK/JUN pathway and the activation of ERK1/2, PI3K/AKT/mTOR signaling, supporting BHB treatment adjunctive nutritional therapy for the treatment of chronic demyelinating diseases, such as multiple sclerosis (MS).

## Introduction

Aging, along with its associated ailments, including cancer, cardiovascular, diabetes, hypertension and neurodegenerative disorders, is a primary public health concern. Multiple sclerosis (MS) is a chronic degenerative neurological disease characterized by autoimmune inflammation, demyelination and axonal degeneration (Liu et al., 2020; Zhang et al., 2020) MS is the main cause of non-traumatic neurological disability in young adults (Klein et al., 2018). Although the disease patterns of MS are different, aging is a critical factor in the pathological progress of MS and increasing disability (Manouchehrinia et al., 2017; Klein et al., 2018).

Ketogenic diet (KD) has been widely used to treat neurodegenerative diseases, including Pelizaeus-Merzbacher disease, Alzheimer’s disease (AD), Parkinson’s disease (PD) and MS (Stumpf et al., 2019; Pavón et al., 2021). Studies have shown that a KD elevates the levels of BHB, improving many age-related neurological diseases, such as AD and PD (Han et al., 2020). BHB, has recently been considered not only as an energy support for cellular requirements, but also as signaling molecule that modulates oxidative stress, inflammatory responses, epigenetics (such as histone methylation and acetylation), RNA-binding proteins and G protein-coupled receptors (Stumpf et al., 2019; Wang et al., 2021). Exogenous BHB has been reported to suppress pathological microglial activation by inhibiting the NLRP3 inflammasome (Wang et al., 2021). In addition, exogenous BHB improved stem cell homeostasis by activating Notch signaling, which is a key signaling axis of tissue regeneration. Thus, BHB can be considered as an important mediator with regenerative potential of neural tissues.

The CPZ mouse model, mimicking the central event of MS pathology, exhibits oligodendrocyte apoptosis following the inflammatory response and demyelination (Zhang et al., 2020). These characteristics make the CPZ model a helpful tool to study primary oligodendrocyte loss and to identify the potential therapeutic targets for demyelination therapy in humans. Activated astrocytes and microglia produce the brain-derived neurotrophic factor (BDNF) during demyelination to promote OL differentiation and survival. BDNF/tropomyosin-related kinase B (TrkB) intracellular signaling is critical for neuronal growth, plasticity, and survival (Sha et al., 2018). Moreover, the BDNF/TrkB signaling pathway is known to stimulate myelin regeneration after demyelination, and is suggested to be used as a therapeutic agent for demyelinating diseases, including stroke and MS (Salehi et al., 2013; Fletcher et al., 2018). It has been reported that inhibition of HDAC3 can promote oligodendrocyte precursor cells (OPCs) to successfully transform into OLs, accelerate remyelination and delay demyelinating injury (Ding et al., 2020). RGFP966, a specific inhibitor of HDAC3, improves functional recovery after spinal cord injury (SCI) by dampening inflammatory cytokines (Kuboyama et al., 2017). Sox2 is expressed in OPCs, which can effectively promote remyelination, and seems to be necessary for the differentiation and development of hippocampal neurons (Peltier et al., 2011; Zhao et al., 2015). Platelet-derived growth factor receptor alpha (PDGF-Rα) is suggested to promote the proliferation and migration of OPCs (An et al., 2020). Moreover, monocarboxylate transporter 1 (MCT1) co-localizes with myelinating oligodendroglia, which are critical for the survival of axons in the central nervous system and support the transport of lactate from oligodendroglia to axons (Lee et al., 2012). In the first 3 weeks after CPZ induction OL apoptosis is strongly regulated by caspase-3. Then, reduction in the activity of caspase-3 was seen in late stages of CPZ intoxication, while the activity of poly ADP-ribose polymerase (PARP) increases, eventually leading to cell dysfunction and death *via* activating apoptosis-inducing factor (AIF; Vega-Riquer et al., 2019).

Another protein playing a role in MS and as cellular stress sensor is transient receptor potential ankyrin 1 (TRPA1), a non-selective cation channel. Recent studies have reported the expression of TRPA1 in glial cells, including astrocytes and oligodendrocytes (Bölcskei et al., 2018). There is considerable evidence suggesting that TRPA1 contributes to increased resting Ca^2+^ levels in astrocytes and long-term potentiation (Shigetomi et al., 2011, 2013). Some studies indicate that TRPA1 deficiency exerts neuro-protection in the CPZ model, which is characterized by preventing anxiety and depressive-like behavior, attenuating demyelination, reducing the apoptosis of mature oligodendrocytes, as well as inhibiting astrocyte and microglia activation (Shigetomi et al., 2013; Sághy et al., 2016). Another *in vivo* study on an Alzheimer’s disease mouse model suggests that TRPA1 mediates in Aβ-induced inflammatory responses of astrocytes and contributes to AD development (Lee et al., 2016). A report by Bosson et al. shows that astrocyte Ca^2+^ hyperactivity contributes to early Aβ toxicity, which involves TRPA1 channels and is associated with CA1 neuron hyperactivity (Bosson et al., 2017), while blocking of TRPA1 channels can reduce myelin damage during the energy deprivation following ischaemia or hypoxia in MS (Davies et al., 2013; Hamilton et al., 2016).

The mitogen-activated protein kinase (MAPK) family includes p38 MAPK, extracellular signal regulated kinase (ERK1/2) and c-jun N-terminal kinase (JNK), which are responsible for signal transduction underlying regulation of cellular functions such as cell proliferation, survival and apoptosis (Ahmed et al., 2020). Previous studies demonstrate that CPZ induced apoptosis of OLs was mediated *via* JNK and p38-MAPK pathways (Sághy et al., 2016), and ERK1/2 activation plays a crucial role in the survival of OLs (Domercq et al., 2011). Sághy et al. (2016) reported that TRPA1 deficiency plays a neuro-protective role in CPZ-induced demyelination by inhibiting the p38-MAPK/JUN pathway and by activating the ERK1/2 pathway, reducing OLs apoptosis and promoting the survival of OLs. Inhibition of poly (ADP-ribose) polymerase (PARP) reduces demyelination in the CPZ model by suppressing p38-MAPK and JUN activation and increasing the activation of the cytoprotective phosphatidylinositol-3 kinase-Akt pathway (Veto et al., 2010). In addition, PI3K/AKT/mTOR signaling plays important roles in OPCs proliferation, differentiation and development of healthy myelin (Mammana et al., 2018).

In this study, we observed the therapeutic potential of BHB treatment in ameliorating anxiety, as well as alleviating memory and learning deficits in CPZ-intoxicated mice. The mechanistic studies showed that BHB inhibited demyelination by up-regulating the expression of BDNF, CNTF and SOX2, down-regulating the expression of TRPA1 and PARP1, inhibiting the p38-MAPK/JNK/JUN pathways, and activating ERK1/2 and PI3K/AKT/mTOR signaling. Thus, our data show that BHB could act as a promising therapeutic agent for chronic demyelinating diseases, such as MS.

## Methods

### Animals

C57BL/6 male mice (8-week-old, weighing 18-20 g) were purchased from Jinan Pengyue Experimental Animal Company, Jinan, China. This study was approved by the laboratory animal ethics committee of Shangdong University. All animals received human care in compliance with the guidelines outlined in the National Institutes of Health guide for the care and use of laboratory animals (NIH Publications No. 8023, revised 1978). Standard rodent pellets food and tap water were available *ad libitum*. The animals were housed under standard laboratory conditions (12:12 h light/dark cycle) for 1 week prior to experimental manipulation.

### CPZ-induced demyelinating model and BHB treatment

**Figure 1:**
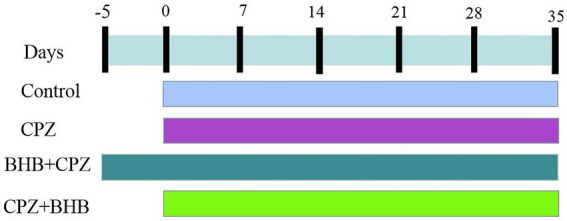
Study time-line of the present study. Mice in the BHB + CPZ group were injected intraperitoneally with BHB (three times per day) prior to initiation of CPZ-feeding. Mice in CPZ + BHB group were intraperitoneally treated with BHB (three times per day), when those animals were fed with CPZ. Body weight was recorded from the beginning of the study to the end, a total of seven time points.

C57BL/6 male mice were fed with standard rodent chow diet with 0.2% (w/w) CPZ (Sigma-Aldrich Inc., St. Louis, MO, USA) for 5 weeks (35 days). Animals were randomly divided into 4 groups (*n* = 10/group) as follows: (A) control group, mice were fed a standard rodent chow diet; (B) CPZ group, mice were fed with a standard rodent chow diet with CPZ and injected with normal saline; (C) BHB + CPZ group, which were intraperitoneally (i.p.) injected with BHB, starting 5 days before CPZ administration; (D) CPZ + BHB group, which were i.p. injected with BHB, starting at CPZ administration for consecutive 35 days until the end of the experiments. BHB (^#^298360, Sigma, USA, dissolved in normal saline) was administered to mice at a dose of 20 mmol/kg three times per day. Animals were euthanized, CC tissues were quickly dissected out from the mouse brains. Blood samples were collected from the tail, and blood ketone levels were measured using a handheld ketone meter (Blood ketone body tester, T-1, Beijing Yicheng bioelectronics Co., Ltd. Beijing, China). The study time-line with milestones is reported in Figure 1.

### Behavioral assessment

#### Open field test

OFT was performed using the same protocol as previously described (Zhang et al., 2020). Anxiety level was measured in an open field square chamber (45 cm width × 45 cm length × 30 cm height). The travel paths of each animal were recorded for 10 min. Measurements included time spent in the central area and traveled distance (total and center) that were recorded by the video-tracking program (BW-OF302, Shanghai Biovill Co., Ltd., China).

#### Morris water maze (MWM)

The MWM test was used to evaluate spatial learning and memory (Zhang et al., 2021). This test consists of three parts: a conditioning test (1 day), a spatial acquisition test trial (5 days), and a probe trial test (1 day). MWM consists of a circular pool (120 cm diameter, 40 cm deep), filled with water at 22 ± 2°C. A black curtain surrounded the pool and contained distinctive marks as visual cues. The pool was divided into four equally sized quadrant: northeast (NE), southeast (SE), southwest (SW) and northwest (NW). The escape platform was placed in quadrant SE (2 cm below the surface of water). Each mouse was placed in the pool in the four different quadrants to find the escape platform. The mouse training time was set to 120 s and mice remained on the platform for 15 s. If a mouse could not find the platform within 120 s, it was gently guided to the platform for 30 s. The probe trial test was evaluated on the 7^th^ day and the escape platform was removed from the pool. The escape latency, total distance swum, speed and number of times crossing the platform were recorded using ANY-maze software.

### Cytokine ELISA assay

The levels of cytokines IL-1β, IL-6 and TNF-α in mouse brains were determined using ELISA assay kits (IL-1β: Beyotime Biotechnology, PI301; IL-6: Beyotime Biotechnology, PI326; TNF-α: Beyotime Biotechnology, PT512) following the manufacturer’s instructions. The results were expressed as pg./mL.

### Luxol fast blue/Cresyl violet (LFB/CV) staining

Brian tissues were incubated with 0.1% luxol fast blue solution (LFB, Sigma-Aldrich) for 24 h at 60°C. Afterwards, brain sections were counterstained with 0.1% cresyl violet solution (BDH, England) and rinsed in distilled water. Myelin fibres appeared blue, nuclei appeared stained in pink and violet. The images were obtained under a light microscope (Olympus, Tokyo, Japan) equipped with a digital camera. The selected fields were chosen from five independent animal slices. The demyelinated areas (LFB staining) were analyzed using ImageJ software (NIH, Bethesda, USA).

### Ultrastructural analysis

C57/BL6 male mice (*n* = 3/group) were anesthetized by intraperitoneal injection of 4% chloral hydrate (400 mg/kg) and fixed by perfusion (4% paraformaldehyde). The CC of mice brains were dissected (tissue blocks of approximately 1 mm^3^) and quickly immersed in 2% glutaraldehyde for 2 hours, then washed with 0.1 M sodium dimethyl arsenate solution for 2 hours, soaked by 1% osmium tetroxide for 2 hours, and sodium dimethyl arsenate solution for 15 min. Then the tissue samples were dehydrated and embedded in epoxy resin (Kim et al., 2018; Shao et al., 2020). The ultrastructure of corpus callosum myelin was examined *via* transmission electron microscope (TME, Hitachi H-7650).

### Histological staining

#### Nissl staining

Neuronal survival was assessed with Nissl staining. The brain sections are stained with Nissl staining solution (0.1% cresyl violet) for 10 min, then rinsed with double distilled water for 1 min. Then dehydration of samples with various concentrations of alcohol was performed, sections were transparentized with xylene for 3 min and sealed with neutral resin for observation (Liu et al., 2020).

#### He staining

HE staining was performed using a HE Staining Kit (G1120, Solarbio). Briefly, staining of brain sections was carried out using Mayers hematoxylin, followed by eosin. Following eosin staining for 50 s, and dehydration by ethanol (95, 100%), the sections were cleared by xylene and mounted. These images were obtained using Nikon’s confocal microscope (Nikon, Japan).

#### Immunohistochemistry staining

The immunohistochemistry was performed as previously described (Zhang et al., 2020). Paraffin-embedded sections of mice brains were stained using an anti-myelin basic protein antibody (MBP, 10458-1-AP, 1:500, Proteintech), GFAP (16825-1-AP, 1:800, Proteintech), Iba-1 (ab178846, 1:500, Abcam), Doublecortin (DCX, ab18723, 1:5,000, Abcam), NeuN (ab177487, 1:500, Abcam), secondary antibody was goat anti-rabbit IgG conjugated with alkaline phosphatase (ab6721, 1:500, Abcam). Vectastain® ABC Kit (Vector Laboratories) was used for immunohistochemical staining. Images were randomly selected from five independent animals. The positive MBP areas were analyzed using ImageJ software (NIH, Bethesda, USA).

#### Immunofluorescence staining

The paraffin-embedded brain tissues were processed for immunofluorescent staining as reported previously (Liu et al., 2020). The sections were incubated with MCT1 antibody (20139-1-AP, 1:800, Proteintech) and APC antibody (P25054, 1:200, Cusabio) at 4°C overnight, and secondary antibodies conjugated with Alexa Green 488 (1:1,000; cat. no. A-11001; Molecular Probes) or with Cy3-labelled secondary antibodies (1:1,000; cat. no. 111–136-144; Jackson ImmunoResearch Laboratories) were used as a secondary antibody for 1 h at room temperature. The slides were visualized by Nikon Eclipse Ti-E microscope.

#### Immunofluorescence double-labeling

Double immunofluorescence staining was performed as previously described (Liu et al., 2020).

Briefly, antigen retrieval was performed in sodium citrate buffer at pH 6, and sections were incubated in blocking solution containing 20% goat serum for 2 h. Then, the paraffin sections were incubated with primary antibodies overnight at 4°C. Primary antibodies were as follows: PDGF-Rα (rabbit, ab234965, 1:200, Abcam), GFAP (rabbit, 16,825-1-AP, 1:800, Proteintech), SOX2 (mouse, ab79351, 1:200, Abcam), TRPA1 (rabbit, 19,124-1-AP, 1:300, Proteintech), Oligo2 (mouse, 66,513-1-Ig, 1:300, Proteintech), and GFAP (mouse, ab4648, 1:50, Abcam). Secondary antibodies were goat anti–rabbit IgG-FITC, Texas red dye-conjugated goat anti-rabbit IgG, goat anti–mouse IgG-FITC, and Texas red dye-conjugated goat anti-mouse IgG (Jackson Immunology) and sections were incubated in the dark for 1.5 h at room temperature. Nuclei were counter-stained with DAPI at 0.5 μg/ml. The images were captured by Nikon Eclipse Ti-E microscope.

### Western blot analysis

The protein of the corpus callosum in mice brains were extracted using the MinuteTM Total Protein Extraction Kit (Invent) supplemented with phosphatase inhibitor and protease inhibitor cocktail (1:100; Sigma). The protein concentration was determined by using the BCA method (BCA Protein Assay kit, Sheng gong® Sangon Biotech). Equal amounts of sample proteins (25 μg) were separated *via* SDS-PAGE (10% or 12% polyacrylamide gels). Subsequently, the proteins were transferred onto polyvinylidene difluoride membranes (Millipore Co, Billerica, MA, USA). After washing, the membranes were blocked for 1.5 h at 37°C with a 5% skim milk solution in TBS-T followed by incubation with primary antibodies at 4°C overnight. Membranes were washed again and then incubated for 1.5 h with horse-radish peroxidase coupled secondary antibodies. Primary antibodies were as follows: PARP1 (ab191217, 1:1,000, Abcam), AIF (ab32516, 1:1,000, Abcam), NG2 (ab275024, 1:1,000, Abcam), SOX2 (11,064-1-AP, 1:1,000, Proteintech), TRPA1 (19124-1-AP, 1:1,000, Proteintech), BDNF (ab108319, 1:2,000, Abcam), TrkB (13129-1-AP, 1:1,000, Proteintech), CNTF (ab270992, 1:1,000, Abcam), p44/42 MAPK (Erk1/2; 9,102, 1:1000, Cell Signaling Technology), phospho-p44/42 MAPK (Erk1/2; 4370S, 1:2,000, Cell Signaling Technology), p38 MAPK (8,690, 1:1,000, Cell Signaling Technology), phospho-p38 MAPK (4511S, 1:1,000, Cell Signaling Technolog), JNK (9,258, 1:1,000, Cell Signaling Technolog), phospho-SAPK/JNK (81E11, 1:1,000, Cell Signaling Technolog), c-Jun (9,165, 1:1,000, Cell Signaling Technolog), Phospho-c-Jun (3,270, 1:1,000, Cell Signaling Technolog). ATK (60203-2-Ig, 1:5,000, Proteintech), phospho-ATK (4,060, 1:2000, Cell Signaling Technolog), PI3 kinase p110α (4,249, 1:1,000, Cell Signaling Technolog), phospho-PI3 Kinase p85 (4,228, 1:1,000, Cell Signaling Technolog), mTOR (2,983, 1:1000, Cell Signaling Technolog), phospho-mTOR (2,971, 1:1,000, Cell Signaling Technolog), HDAC3 (10255-1-AP, 1:2,000, Proteintech), and β-actin (20536-1-AP, 1:5,000, Proteintech). The secondary antibodies were as follows: goat anti-rabbit IgG conjugated to horseradish peroxidase (ab6721, 1:10,000, Abcam). After washing with PBST, the immunoreactive protein bands were visualized using chemiluminescence (ECL) substrate with the UVP digital image system (UVP, USA).

### Statistical analysis

SPSS statistics 17.0 software was used for statistical analysis. One-way analysis of variance (ANOVA) determined differences between treatments, followed by the Bonferroni post-hoc test. The data is expressed as means ± SEM. *p* < 0.05 was considered statistically significant.

## Results

### BHB dose–response curve and body weights

**Figure 2:**
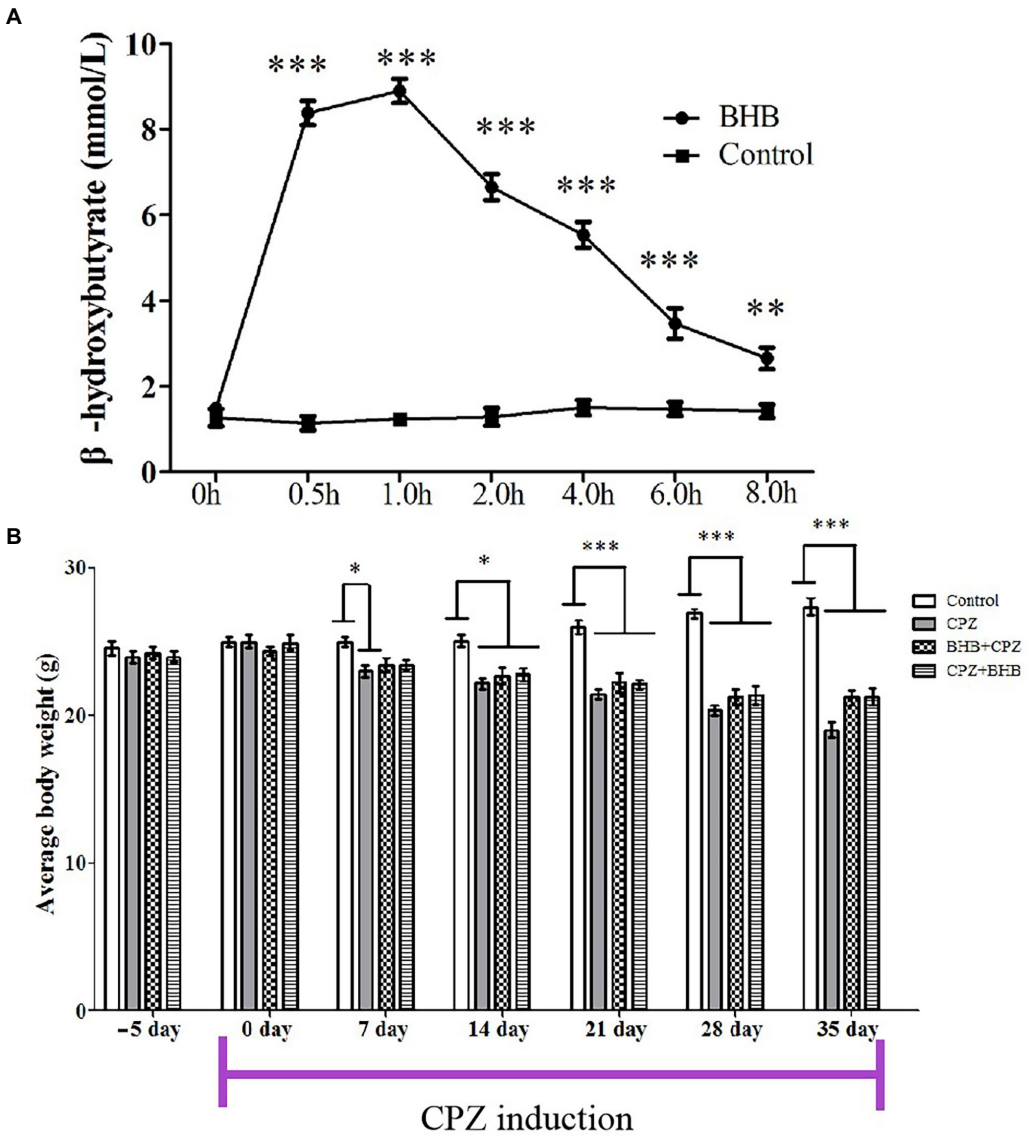
Dose–response curves of blood β-hydroxybutyrate concentrations after intraperitoneal injection of BHB **(A)** and average body weights of mice at each experimental time point **(B)**. *N* = 10 mice/group. The data were presented as mean. *p < 0.05, **p < 0.01 and ***p < 0.001 versus the control group.

The blood BHB concentration in BHB-treated mice was significant higher than that in control mice, which confirms that exogenous BHB supplementation could significantly elevate the concentration of β-hydroxybutyrate in the blood (Figure 2A) and brain (Supplementary data 1). In the present study, the blood BHB concentrations were 8.38 ± 0.28 mmol/l (***p < 0.001) at 0.5 h, 0.80 ± 0.28 mmol/l (***p < 0.001) at 1 h, 6.65 ± 0.30 mmol/l (***p < 0.001) at 2 h, 5.53 ± 0.30 mmol/l (***p < 0.001) at 4 h, 3.47 ± 0.36 mmol/l (***p < 0.001) at 6 h, and 2.65 ± 0.25 mmol/l (**p < 0.01) at 8 h after administration. The body weight of mice in all experimental groups was similar to average value, there was no statistical difference among the groups (control: 24.97 ± 0.32 g; CPZ: 24.97 ± 0.44 g; BHB + CPZ: 24.56 ± 0.54 g; CPZ + BHB: 24.9 ± 0.52 g). The body weight in the control group increased gradually over time, while it decreased in the CPZ group. At the end of the study, average body weight was 27.37 ± 0.57 g for control mice, 19.02 ± 0.50 g for CPZ-fed mice, 21.51 ± 0.51 g for BHB + CPZ-fed mice, and 21.25 ± 0.58 g for CPZ + BHB mice. Although body weights of mice tended to increase in the BHB + CPZ and CPZ + BHB groups compared to the CPZ group, no significant differences between groups were observed at the end of the experiment (*p* = 0.056, BHB + CPZ vs. CPZ; *p* = 0.050, BHB + CPZ vs. CPZ; Figure 2B).

### Behavioral tests

#### OFT test

**Figure 3:**
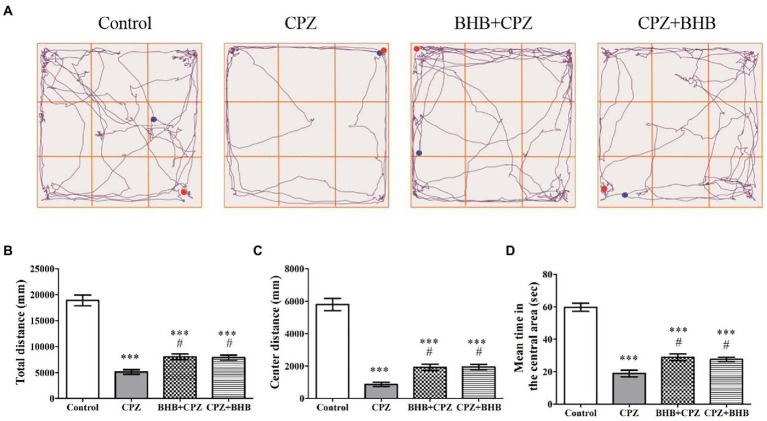
OFT behavioral assessment. **(A)** Representative images show typical examples of mice trajectories in the OFT at 35 days after CPZ induction; blue dots represent the starting point and red dots represent the final point. Total distance traveled **(B)**, distance traveled in the central zone **(C)**, and time spent in the central area **(D)** were compared among control, CPZ, BHB + CPZ and CPZ + BHB groups. Data are presented as mean ± SEM. *N* = 10 mice/group. ***p < 0.001 versus the control group; ^#^*p* < 0.05 versus the CPZ group.

OFT test was performed on mice to evaluate the anxiety-like behavior (Wang et al., 2018). A remarkable decrease in the distance traveled (total distance and distance traveled in the central area, ***p < 0.001 vs. control group), and time spent in the central area (***p < 0.001 vs. control group) were observed in the CPZ group (Figures 3A–D). These results suggest that mice in the CPZ group have increased anxiety-like behaviors. Upon treatment with BHB, the distance moved (total distance and distance traveled in the central area, ***p < 0.001 vs. control group), and time spent in the central area (***p < 0.001 vs. control group) were both increased in BHB + CPZ and CPZ + BHB groups (Figures 3B–D), suggesting that BHB pre-intervention or simultaneous intervention can decrease the anxiety-like behavior of CPZ-fed mice.

#### MWM test

**Figure 4:**
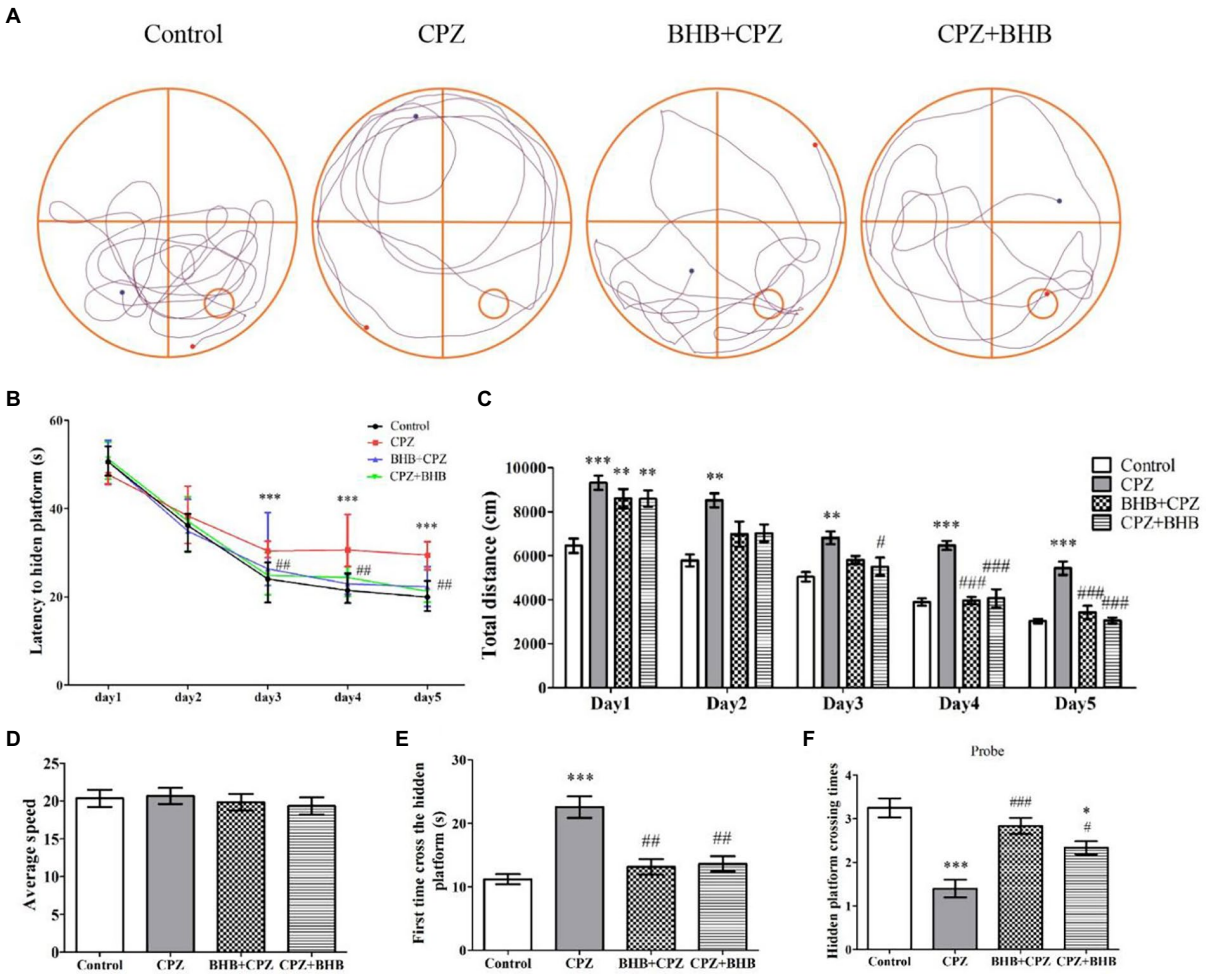
Effect of BHB on memory impairment in CPZ-fed mice. **(A)** Representative mouse search paths on the 6^th^ day after starting with the MWM experiment, blue dots represent the starting point and red dots represent the final point. The red square indicates the platform location and the blue square indicates the start location. **(B)** Escape latency of the control, CPZ, BHB + CPZ and CPZ + BHB groups over five-day training. **(C)** Total distance traveled during training days and **(D)** average speed among the four groups were recorded (mm/s). **(E)** First time to cross the hidden platform and **(F)** the hidden platform crossing times among the four groups. Data are presented as mean ± SEM. *N* = 10 mice/group. *p < 0.05, **p < 0.01, and ***p < 0.001 versus the control group; ^#^*p* < 0.05, ^##^*p* < 0.01, and ^###^*p* < 0.001 versus the CPZ group.

To investigate whether BHB can ameliorate the deficits in spatial learning and memory in CPZ-fed mice, the MWM test was carried out. Representative trajectory paths are shown in Figure 4A. Images show that the CPZ-fed mice needed mor time to find the platform than the mice in the control group and BHB treatment group. The mice in the CPZ group showed a longer escape latency on the 3rd (30.63 ± 1.59 s), 4th (31.63 ± 4.64 s) and 5th (29.25 ± 2.17 s) days compared with the control group (the 3rd day, 23.65 ± 3.5 s; the 4th day, 21.85 ± 2.72 s; the 5th day, 20.03 ± 2.55 s; Figure 4B), while mice in the BHB + CPZ and CPZ + BHB showed a shorter escape latency on the 3rd (BHB + CPZ, 27.78 ± 5.85 s; CPZ + BHB, 24.73 ± 3.23 s), 4th (BHB + CPZ, 23.75 ± 3.02 s; CPZ + BHB) and 5th (BHB + CPZ, 21.12 ± 3.66 s; CPZ + BHB, 22.10 ± 3.07 s) days compared to the CPZ group (Figure 4B). The traveled total distance in all four groups of mice showed a decreased tendency (Figure 4C). Compared to the control group, mice in the CPZ group traveled longer distances from day 1 to day 5. On the 4th and 5th day, the total traveled distances in the BHB + CPZ and CPZ + BHB groups were shorter than those in the CPZ group (Figure 4C). No differences in average travel speeds were detected among the four groups (*p* > 0.05; Figure 4D). Moreover, the CPZ-fed mice took longer to cross the hidden platform for the first time (*p* < 0.001 vs. control group, Figure 4E) and the number of platform crossings was lower than that of the control group (*p* < 0.001 vs. control group, Figure 4F). However, mice spent a shorter time to cross the hidden platform in the BHB + CPZ group and CPZ + BHB group than that in the CPZ group for the first time (*p* < 0.01 BHB + CPZ vs. control group; p < 0.01 CPZ + BHB vs. control group, Figure 4E), and crossed the platform location more times in the BHB + CPZ group and CPZ + BHB group than that in the CPZ group (*p* < 0.001 BHB + CPZ vs. control group; *p* < 0.05 CPZ + BHB vs. control group, Figure 4F), suggesting that BHB treatment can improve the spatial learning and memory abilities in CPZ-fed mice.

### BHB improves demyelinating lesion in CPZ-Fed mice

**Figure 5:**
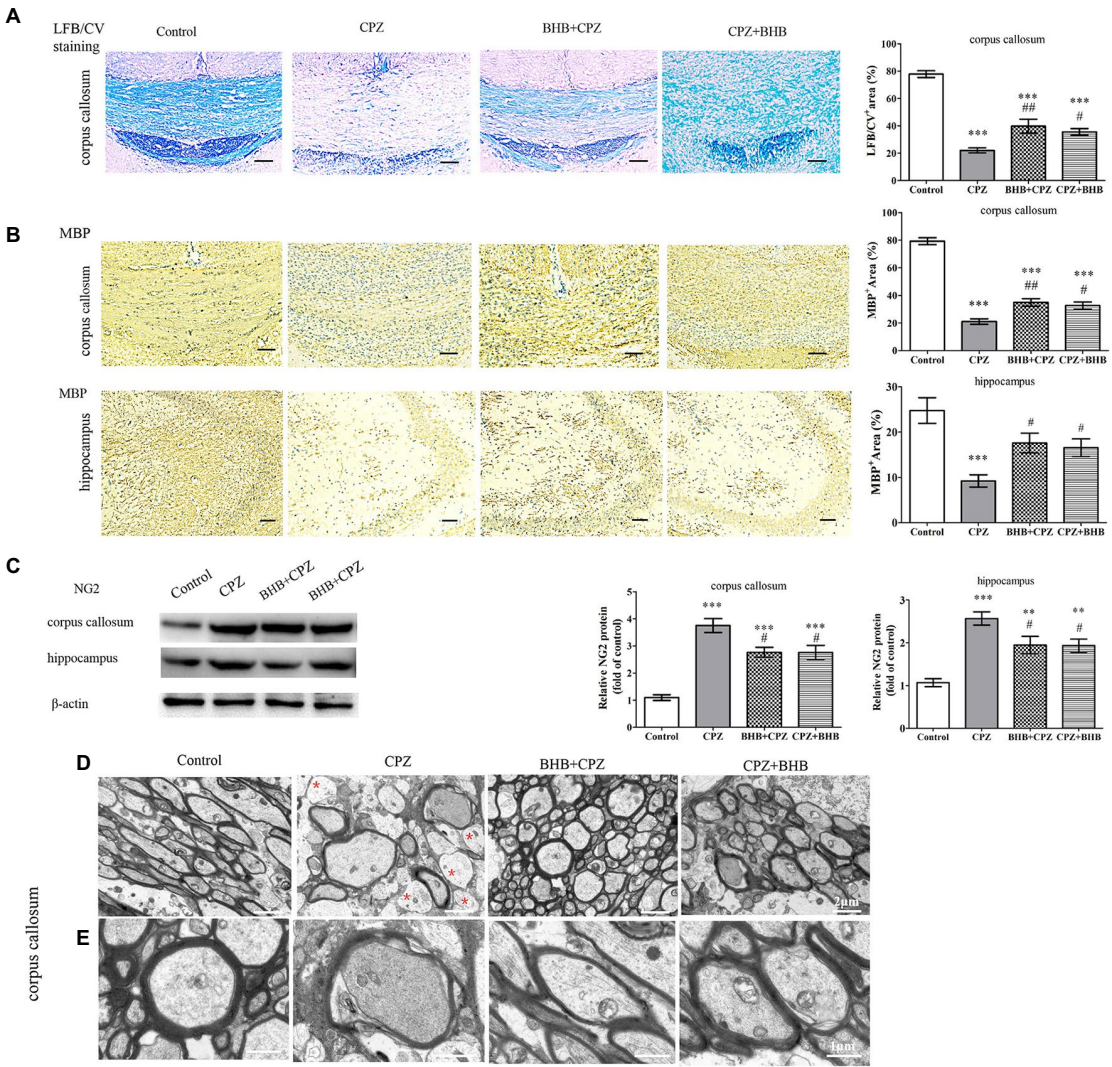
BHB treatment reduced demyelination in CPZ-fed mice. **(A)** Histological evaluation of demyelination by LFB/CV staining. Scale bars = 50 μm. **(B)** Representative MBP staining and intensity quantification in the corpus callosum and hippocampus of CPZ-fed mice among different groups. Scale bars = 50 μm. **(C)** Western blot analysis of NG2 expression in CPZ-fed mice. (D and E) Representative TEM images of demyelination in the corpus callosum of CPZ-fed mice. Note that non-myelinated axons (red asterisks) are frequent in CPZ-fed mice; D magnified 4,000×, scale bars = 2 μm. E magnified 10,000×, scale bars = 1 μm. Data are presented as mean ± SEM. *N* = 3 per experimental group; experiment repeated two times. **p < 0.01 and ***p < 0.001 versus the control group; ^#^*p* < 0.05 and ^##^*p* < 0.01 versus the CPZ group.

Histologically, demyelination lesions were revealed by the LFB/CV stain. CPZ-fed mice revealed significantly lower LFB/CV staining indensity in the corpus callosum (Figure 5A; *p* < 0.001). The LFB/CV level was significantly increased in BHB + CPZ group and CPZ + BHB group, when compared with the CPZ group (BHB + CPZ group vs. CPZ group, *p* < 0.01, Figure 5A; CPZ + BHB group vs. CPZ group, *p* < 0.05, Figure 5A). Moreover, we used the MBP immunohistochemical staining to further confirm the extent of demyelination. MBP staining showed the loss of MBP-positive fibers in the corpus callosum of CPZ-fed mice (CPZ group vs. control group, *p* < 0.001, Figure 5B), indicating severe demyelination. However, BHB + CPZ-fed mice and CPZ + BHB mice both show increased intensity of myelin staining by MBP in the corpus callosum, as compared to CPZ-fed mice (BHB + CPZ group vs. CPZ group, *p* < 0.01, Figure 5B; CPZ + BHB group vs. CPZ group, *p* < 0.05, Figure 5B). In the hippocampus, a similar phenomenon was observed. MBP immunostained areas of CA3 sub-regions were significantly increased in BHB + CPZ-fed mice and CPZ + BHB mice, when compared with the CPZ-fed mice (BHB + CPZ group vs. CPZ group, *p* < 0.05, Figure 5B; CPZ + BHB group vs. CPZ group, *p* < 0.05, Figure 5B). These results suggest demyelination lesions were significantly reduced by BHB treatment in CPZ-fed mice.

NG2, a marker of oligodendrocyte precursor cells, was dramatically increased in the CPZ-fed mice (Zhang et al., 2020). In the present study, Western Blot results showed that the NG2 expression was higher in the corpus callosum and hippocampus in CPZ-fed mice, while BHB treatment decreased the level of NG2, as compared to CPZ-fed mice (corpus callosum, BHB + CPZ group vs. CPZ group, *p* < 0.05, Figure 5C; corpus callosum, CPZ + BHB group vs. CPZ group, *p* < 0.05; hippocampus, BHB + CPZ group vs. CPZ group, *p* < 0.05; hippocampus, CPZ + BHB group vs. CPZ group, *p* < 0.05, Figure 5C), indicating that proliferation and survival of OPCs were regulated by BHB. In addition, quantitation of myelinated axons within lesion sites of the corpus callosum was conducted by TEM image analyses. It revealed that CPZ-fed mice exhibited abnormal morphology, including demyelination, de-compaction of myelin sheath, swelling of axons, myelin distortion and some loss of myelinated profiles (Figure 5D), while more myelinated axon were observed in the corpus callosum area of BHB + CPZ-fed mice and CPZ + BHB mice vs. CPZ-fed mice (Figure 5D). Both, the increased myelinated axon density and myelin thickness indicated that the structure and function of axons recovered with BHB administration.

### Effects of BHB on microglia and astrocytes in the corpus callosum and hippocampus of CPZ-induced mice

**Figure 6:**
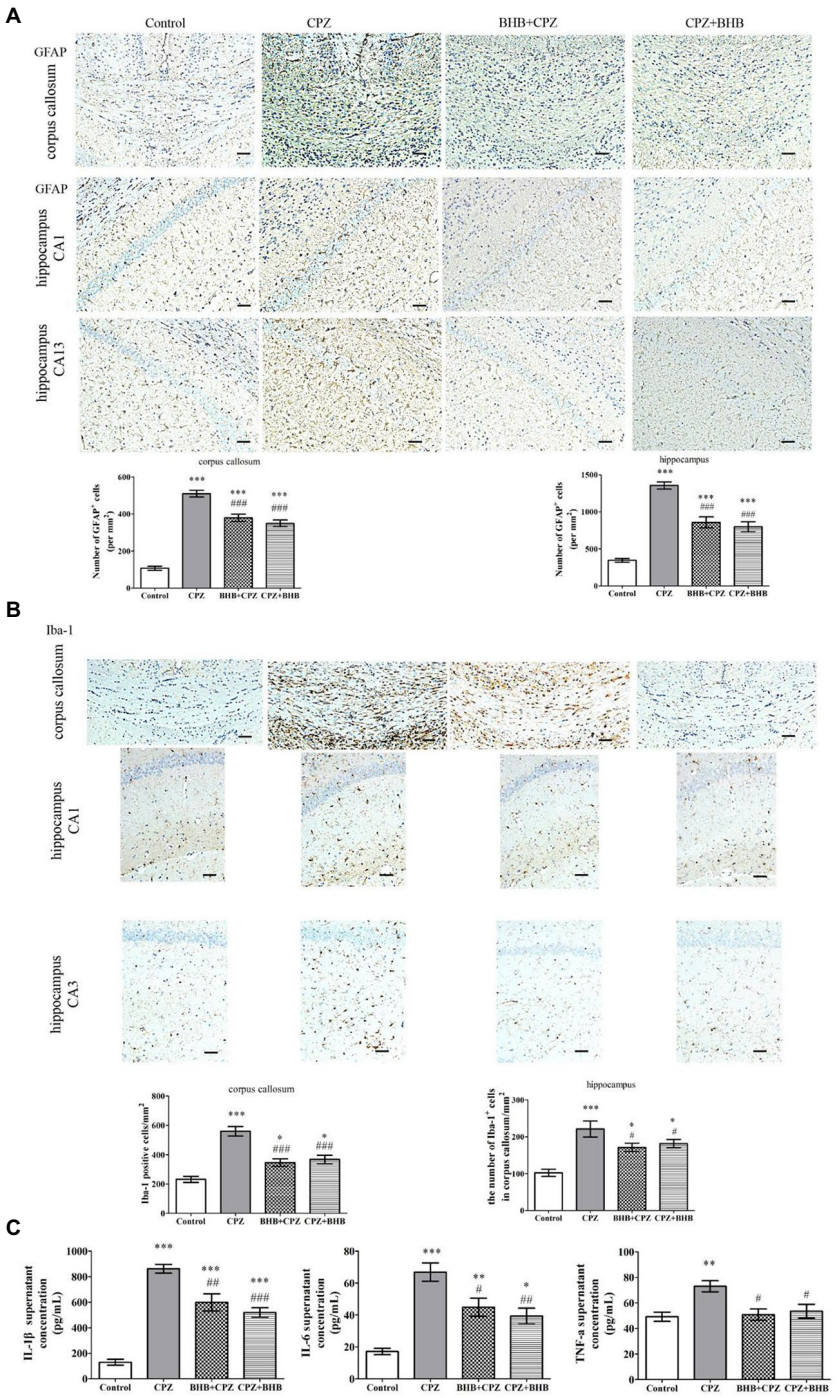
Immunohistochemistry for the microglia and astrocytes in the corpus callosum and hippocampus, and the levels of IL-1β, IL-6, and TNF-α in brains of mice at day 35 after CPZ induction. **(A)** Representative GFAP staining and intensity quantification in the corpus callosum and hippocampus of CPZ-fed mice in different groups. **(B)** Representative Iba-1 staining and intensity quantification in the corpus callosum and hippocampus of CPZ-fed mice in different groups. **(C)** IL-1β, IL-6 and TNF-α levels in brains were analyzed by ELISA. Data are presented as mean ± SEM. *N* = 3 per experimental group; experiment repeated two times. Scale bars = 50 μm. *p < 0.05, **p < 0.01, and ***p < 0.001 versus the control group; ^#^*p* < 0.05, ^##^*p* < 0.01, and ^###^*p* < 0.001 versus the CPZ group.

Increasing evidences suggest that the activation of astrocytes and microglia can aggravate demyelinating lesions in the CPZ model. Astrocytosis and microgliosis were evaluated by GFAP and Iba-1 staining, respectively. In the present study, our results were similar to our previous study on the CPZ-fed mice, significantly elevated expression levels of GFAP-positive astrocytes were noted in the corpus callosum and hippocampus of CPZ-fed mice compared with the control group (*p* < 0.001), whereas a decrease was found in the BHB + CPZ treated mice and CPZ + BHB treated mice (as shown in Figure 6A). It has been reported that over-activation of astrocytes prevents myelin sheath regeneration and causes neuronal damage. Thus, reduction of the extent of astrogliosis might be beneficial for remyelination. In the study, we found that pretreatment or after-treatment with BHB significantly reduced GFAP expression and the morphology of GFAP return to normal conditions, suggesting that BHB treatment makes an environment conducive to normal neuronal growth and remyelination.

Microglia were visualized using Iba-1^+^ immunohistochemical staining. Our previous study indicated that microglia were activated in mice after receiving CPZ, and hyperactivation of microglia could produce proinflammatory cytokines, such as IL-1β, IL-6, and TNF-α, which subsequently induced chronic inflammation and aggravate the process of demyelination (Zhang et al., 2020). In the present study, we investigated the Iba1^+^ microglia in the corpus callosum and hippocampus of mice fed with CPZ. The number of Iba1^+^ microglia in the corpus callosum and hippocampus was significantly increased (corpus callosum, CPZ group vs. control group, *p* < 0.001, Figure 6B; hippocampus, CPZ group vs. control group, *p* < 0.001, Figure 6B), while the number of Iba1^+^ microglia was significantly decreased in corpus callosum and hippocampus sections of CPZ-fed mice after treating with BHB (corpus callosum, BHB + CPZ group vs. CPZ group, *p* < 0.001, CPZ + BHB group vs. CPZ group, *p* < 0.05, Figure 6B; hippocampus, BHB + CPZ group vs. CPZ group, *p* < 0.001, CPZ + BHB group vs. CPZ group, *p* < 0.05, Figure 6B). Moreover, microglia activation was associated with neuroinflammation during CPZ progression. The expression of IL-1β, IL-6 and TNF-α in the brain extract increased markedly in CPZ-fed mice, while the levels of IL-1β, IL-6 and TNF-α decreased in BHB + CPZ treated mice and CPZ + BHB treated mice (^#^*p* < 0.05, ^##^*p* < 0.01 and ^###^*p* < 0.001, Figure 6C). These results suggested that BHB might inhibit the CPZ-induced activation of microglia and decrease inflammatory response.

### Effect of BHB on oligodendrocytes

**Figure 7:**
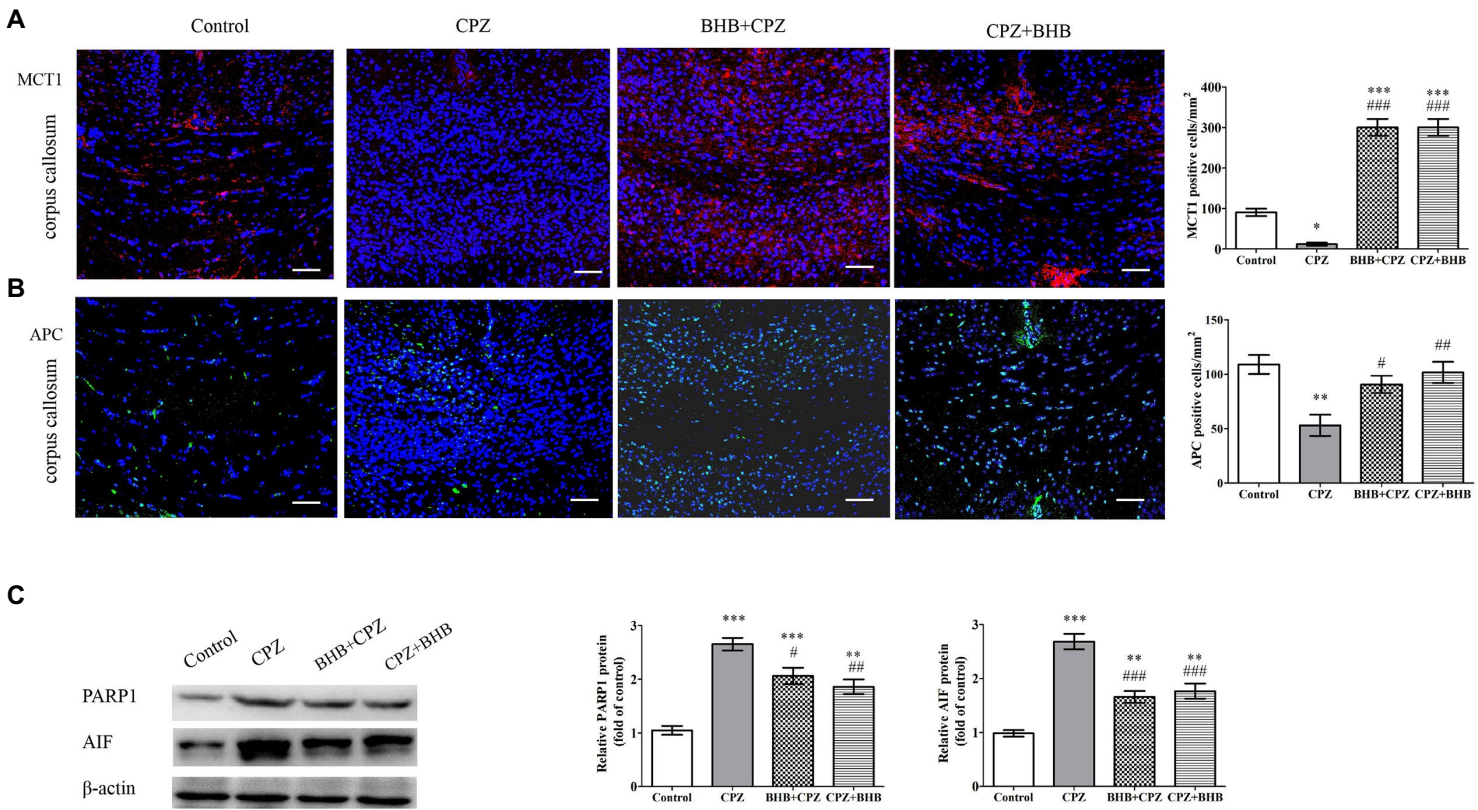
Expression of MCT1 and APC, and PARP1 and AIF in the corpus callosum of CPZ-fed mice. The immunofluorescent staining and intensity quantification of MCT1 **(A)** and APC **(B)** in the corpus callosum of mice in different groups. **(C)** WB assays (PARP1, AIF and β-actin as a loading control). Scale bars = 50 μm. Data are presented as mean ± SEM. *N* = 3 per experimental group; experiment repeated two times. *p < 0.05, **p < 0.01, and ***p < 0.001 versus the control group; ^#^*p* < 0.05, ^##^*p* < 0.01, and ^###^*p* < 0.001 versus the CPZ group.

Lee et al. reported that MCT1 (a transporter for monocarboxylic acids such as lactate and ketone bodies) was highly expressed in oligodendroglia, and the disruption of this transporter can lead to axonal damage and neuronal loss (Lee et al., 2012). Fluorescent immunostaining for MCT1 in the corpus callosum revealed that expression of MCT1 was predominantly decreased in CPZ-fed mice, while the expression of MCT1 was significant increased in BHB + CPZ and CPZ + BHB treated mice (BHB + CPZ vs. control, *p* < 0.001; CPZ + BHB vs. control, *p* < 0.001, Figure 7A). This finding indicates that BHB can create an environment to meet the higher metabolic needs of myelinating oligodendrocytes by increasing the expression of MCT1.

APC immunolabeling was used to evaluate the oligodendrocyte survival after CPZ induction. As shown in Figure 7B, the density of APC positive cells was dramatically decreased in the corpus callosum of CPZ-fed mice as compared with the control group (CPZ vs. control, *p* < 0.001, Figure 7B). In contrast, the densities of APC positive cells were increased in the corpus callosum of mice in BHB + CPZ and CPZ + BHB groups. These results suggest that OLs are vulnerable to CPZ-induced damages, and BHB can increase the survival of oligodendrocytes.

It has been reported that up-regulation of PARP promotes the activation of apoptosis-inducing factor (AIF)-mediated OLG apoptosis in old mice during the later stage of CPZ intoxication (Praet et al., 2014). As shown in Figure 7C, WB assay results showed that CPZ induced increased expression of apoptotic proteins, such as PARP1 and AIF, compared to the control group, whereas the expression of PARP1 and AIF was decreased in BHB + CPZ and CPZ + BHB groups (PARP1, BHB + CPZ vs. CPZ, *p* < 0.05; AIF, BHB + CPZ vs. CPZ, *p* < 0.01; PARP1, CPZ + BHB vs. CPZ, *p* < 0.001; AIF, CPZ + BHB vs. CPZ, *p* < 0.001, Figure 7C). These results demonstrate that CPZ induces OLG apoptosis in the corpus callosum of CPZ-fed mice, which could be attenuated by BHB treatment *via* inhibition of PARP and AIF.

**Figure 8:**
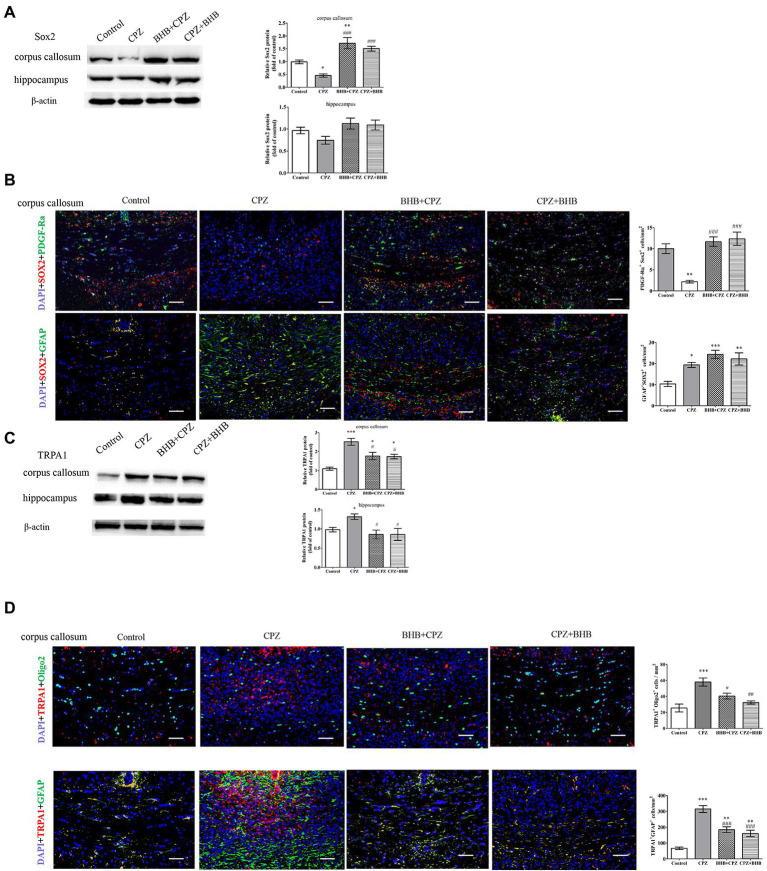
Expression of Sox2 and TRPA1 in the corpus callosum shown by immunofluorescence and WB. WB assays indicate the expression of Sox2 **(A)**, TRPA1 **(C)** and β-actin (loading control) in the corpus callosum and hippocampus of mice in different groups. Double immunofluorescence staining for PDGF-Ra^+^ Sox2^+^ and GFAP^+^ Sox2 ^+^ **(B)**, and oligo2^+^ TRPA1^+^ and GFAP^+^ TRPA1^+^ **(D)** in the corpus callosum of mice. Data are presented as mean ± SEM. *N* = 3 per experimental group; experiment repeated two times. Scale bars = 50 μm. *p < 0.05, **p < 0.01, and ***p < 0.001 versus the control group; ^#^*p* < 0.05, ^##^*p* < 0.01, and ^###^*p* < 0.001 versus the CPZ group.

Moreover, Sox2 is known to regulate the proliferation of oligodendrocytes (He et al., 2021). First, we detected the expression of Sox2 in the corpus callosum and hippocampus, and found that BHB + CPZ and CPZ + BHB treatment increased Sox2 expression in the corpus callosum (BHB + CPZ vs. CPZ, *p* < 0.001; CPZ + BHB vs. CPZ, *p* < 0.001, Figure 8A). In addition, Sox2 expression showed an increased trend in the hippocampus of mice in the BHB + CPZ and CPZ + BHB groups, although it was not significant (Figure 8A). Next, we measured the expression of Sox2 and GFAP by immunofluorescence double staining. The results showed that PDGF-Ra^+^ OPCs and GFAP^+^ astrocytes expressed Sox2 in the corpus callosum of mice (Figure 8B). Moreover, we found that more of the Sox2 deposits co-localized with PDGF-Ra^+^ cells and GFAP ^+^ astrocytes in the mice of the BHB + CPZ group and the CPZ + BHB group when compared to the CPZ group (Figure 8B).

The deletion of TRPA1 could attenuate CPZ-induced demyelination by reducing the apoptosis of mature oligodendrocytes (Sághy et al., 2016). In our experiments, we found that levels of TRPA1 were significantly reduced in the BHB + CPZ group and CPZ + BHB group as compared with the CPZ group (^#^*p* < 0.05, respectively, Figure C). Interestingly, the immunofluorescence double staining showed that both PDGF-Ra^+^ OPCs and GFAP^+^ astrocytes expressed Sox2 (Figure 8D), and the density of TRPA1^+^ oligo2^+^ and TRPA1^+^ GFAP^+^ was reduced in the BHB + CPZ group and CPZ + BHB group than those in CPZ group (^#^*p* < 0.05 and ^###^*p* < 0.001, Figure 8D).

### Morphology of the hippocampus

**Figure 9:**
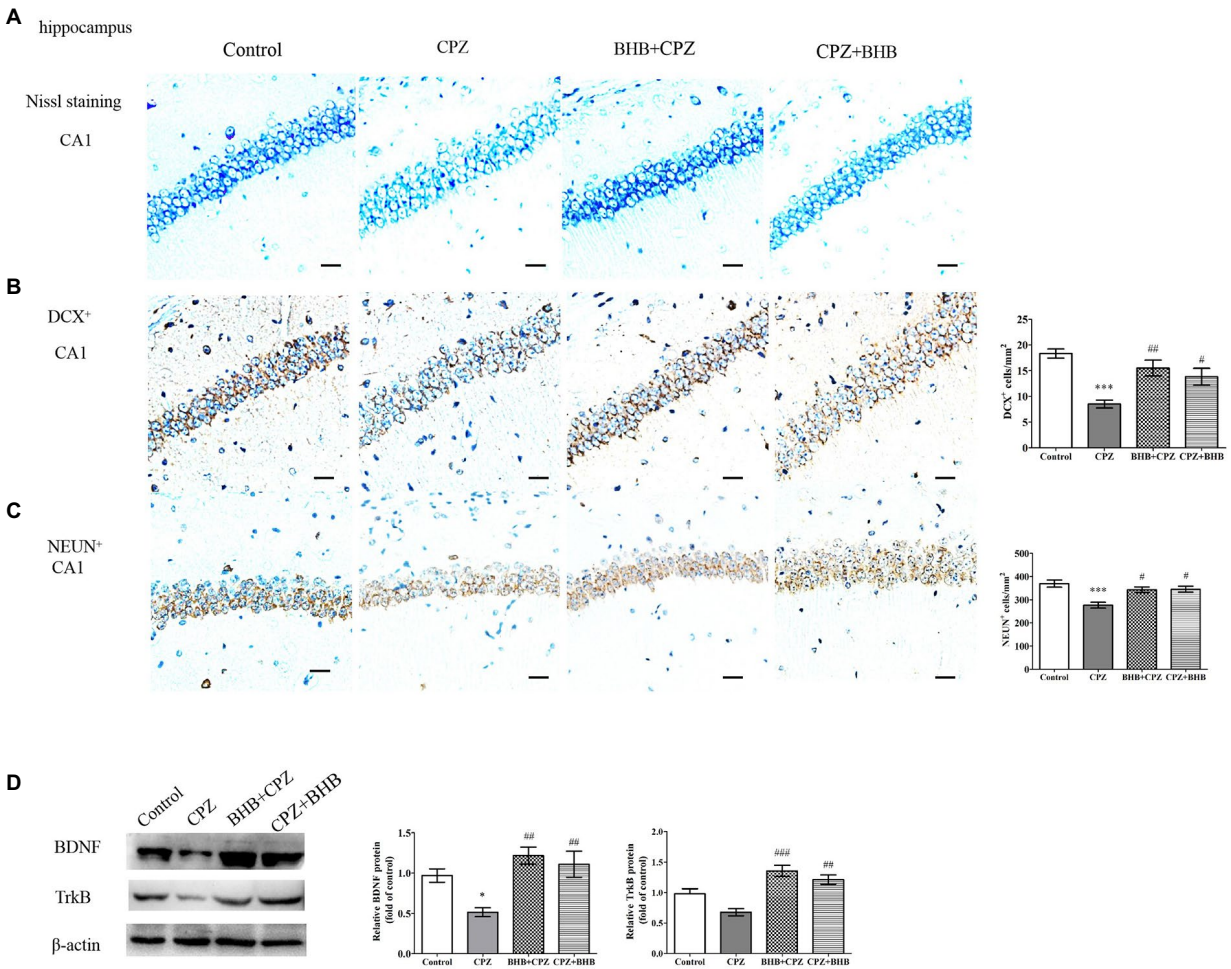
Histological images of the hippocampus and WB analysis of expression of BDNF and TrkB. **(A)** Nissl staining of the CA1 in the hippocampus in different treated groups. Distribution of immunoreactive cells for **(B)** DCX^+^ and **(C)** NeuN^+^ in the hippocampal CA1 of mice after CPZ exposure. The histograms show the number of immunoreactive cells in the different groups. **(D)** Representative WB of BDNF, TrkB and β-actin in hippocampal protein samples and semi-quantitative analysis of WB results; β-actin served as loading control. Bars = 25 μm. Data are presented as mean ± SEM. *N* = 3 per experimental group; experiment repeated two times. *p < 0.05 and ***p < 0.001 versus the control group; ^#^*p* < 0.05, ^##^*p* < 0.01, and ^###^*p* < 0.01 versus the CPZ group.

Next, neuropathological changes were investigated. Figure 9A shows Nissl staining in the hippocampal CA1 regions of mice. These staining revealed that neurons in hippocampal CA1 region contained abundant cytoplasm, and were neatly arranged, demonstrating intact morphology in the control group. Specifically, mice in CPZ group have disorganized neuronal layering, while mice both in the BHB + CPZ and the CPZ + BHB groups displayed a recovering effect of the neuronal damage. In addition, it a decrease in the number of DCX^+^ progenitor cells and neuron-specific nuclear protein (NeuN)^+^ cells in the hippocampus of mice in the BHB + CPZ and the CPZ + BHB groups comparison to control group was observed (*p* < 0.001, CPZ vs. control, respectively, Figures 9B,C). Notably, the density of DCX^+^ cells and NeuN^+^ cells was increased in the hippocampus of mice following BHB + CPZ and CPZ + BHB treatment, compared to that in the brain of CPZ-fed mice (DCX^+^, BHB + CPZ vs. CPZ, *p* < 0.01; DCX^+^, CPZ + BHB vs. CPZ, *p* < 0.05; NeuN^+^, BHB + CPZ vs. CPZ, *p* < 0.05; NeuN ^+^, CPZ + BHB vs. CPZ, *p* < 0.05, Figures 9B,C). Immunohistochemical detection of DCX^+^ (Supplementary data 2) revealed that more DCX^+^ cells in the mouse dentate gyrus subgranular zone in BHB treated groups than those in the CPZ group, indicating that BHB affect neurogenesis. We also found that the protein expression of BDNF in the CPZ group was significantly lower than that in the control group (CPZ vs. control, *p* < 0.05, Figure 9D), while the BHB + CPZ and CPZ + BHB groups exhibited significantly higher expression of BDNF than the CPZ group (*p* < 0.01, respectively, Figure 9D). In parallel with alterations of BDNF levels, its receptor TrkB was also significantly increased in BHB + CPZ and CPZ + BHB groups (BHB + CPZ vs. CPZ, *p* < 0.001; CPZ + BHB vs. CPZ, *p* < 0.01, respectively, Figure 9D). Thus, experimental results suggest that BHB can create an environment that promotes the regeneration of the central nervous system, such as releasing neurotrophic factor (BDNF) and enhancing the TrkB expression, which could promote the production of neuronal cells.

### BHB modulates mitogen-activated protein kinase pathways and PI3K/Akt/mTOR signaling pathway in the corpus callosum of CPZ model

**Figure 10:**
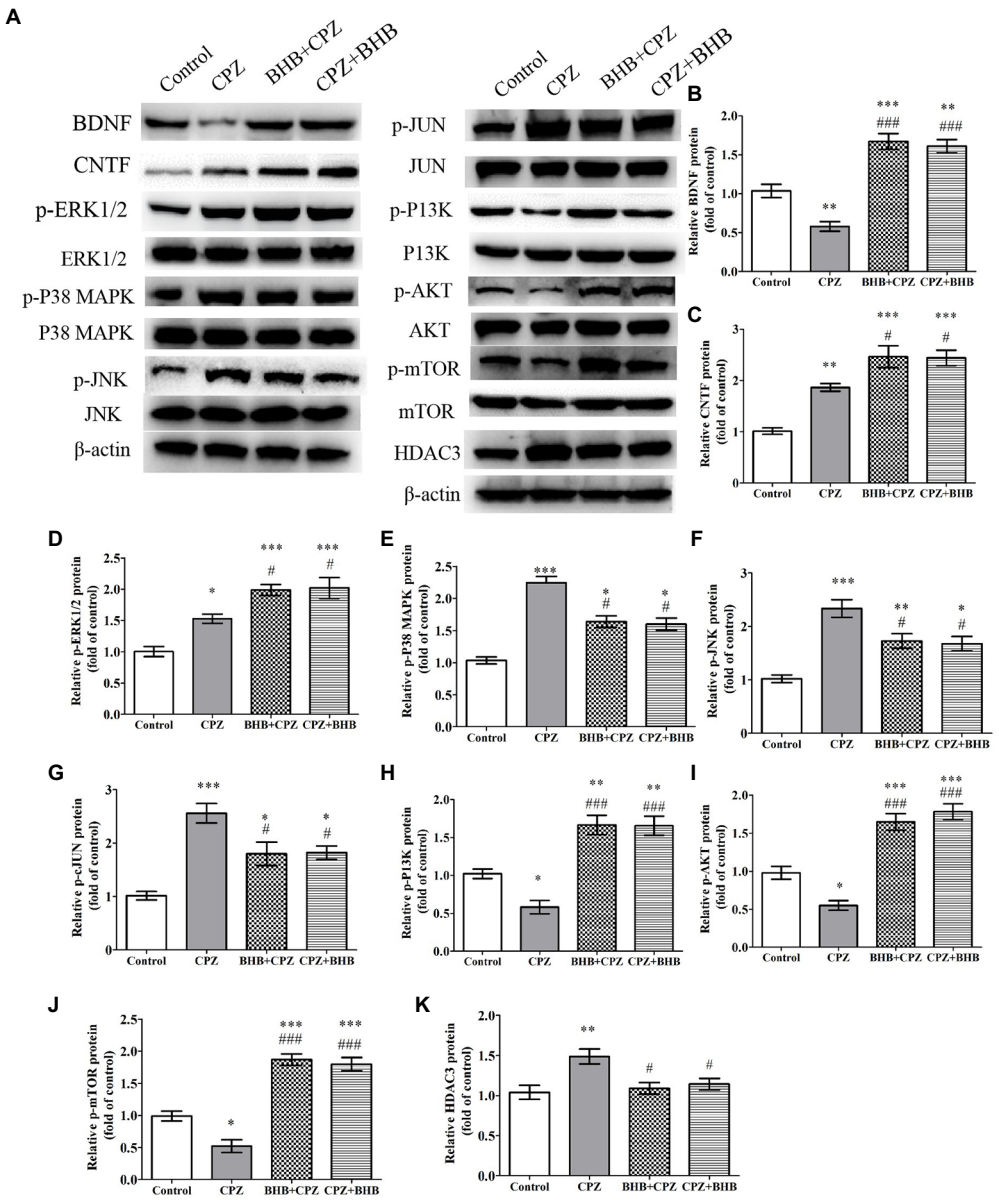
The effect of CPZ on the expression of BDNF CNTF and HDAC3, and the phosphorylation state of ERK1/2, p38-MAPK, JNK, c-Jun, PI3K, Akt, mTOR in the corpus callosum of CPZ-fed mice in different groups. **(A)** Representative images of WBs are shown. WBs were performed to detect the **(B)** BDNF, **(C)** CNTF, **(D)** p-ERK1/2, **(E)** p-P38-MAPK, **(F)** p-JNK, **(G)** p-JUN, **(H)** p-PI3K, **(I)** p-PI3K, **(J)** p-mTOR, and **(K)** HADC3 protein levels in CPZ-fed mice. β-actin was used as an internal control. Quantification of WB bands was conducted by ImageJ. The data are presented as mean ± SEM. *N* = 3 per experimental group; experiment repeated two times. *p < 0.05, *p < 0.01, and ***p < 0.001 versus the control group; ^#^*p* < 0.05 and ^###^*p* < 0.001 versus the CPZ group.

Neurotrophic factors, such as BDNF can promote OPCs survival and maturation, and CNTF is beneficial to axonal sprouting, OLs generation and maturation (Siebert and Osterhout, 2021). In this study, we found that the expressions of BDNF and CNTF were increased in BHB + CPZ and CPZ + BHB groups as compared with the CPZ group (BDNF, BHB + CPZ vs. CPZ, *p* < 0.001, CPZ + BHB vs. CPZ, *p* < 0.001, Figure 10B; CNTF, BHB + CPZ vs. CPZ, *p* < 0.05, CPZ + BHB vs. CPZ, *p* < 0.05; Figure 10C).

It has been reported that CPZ triggers the activation of proapoptotic JNK and p38-MAPK pathways in response to OL death (Sághy et al., 2016). ERK1/ 2 is involved in protecting pre-myelinating oligodendrocytes (PreOLs) and reducing oxidative injury (Cai et al., 2016). Moreover, the PI3K/Akt/mTOR signaling pathway has been confirmed to be involved in the process of remyelination and can increase the number of OLs (Liu et al., 2017). In the present study, CPZ significantly induced the activation of the mitogen-activated protein kinases, phosphorylated p38-MAPK, phosphorylated JNK, phosphorylated ERK1/2, as well as phosphorylated c-Jun when compared with the control group (*p < 0.05, **p < 0.01 and ***p < 0.001, Figures 10D–G). Interesting, BHB treatment decreased phosphorylation levels of p38-MAPK, JNK, c-Jun ^(#^*p* < 0.05) and increased ERK1/2 phosphorylation (^#^*p* < 0.05). Moreover, the levels of the phosphorylated PI3K, phosphorylated Akt and phosphorylated mTOR were dramatically reduced in the CPZ group in comparison to the control group, while they were dramatically increased in the BHB + CPZ and CPZ + BHB groups in comparison to the CPZ group (^###^*p* < 0.001, Figures 10H–J). We also found that the HDAC3 expression was markedly decreased in the BHB + CPZ and CPZ + BHB groups when compared with the CPZ group (^#^*p* < 0.05, Figure 10K).

## Discussion

Aging consider as the ultimate target for prevention of progressive disease course, which is the most important determinant of disability worsening in MS (Zeydan and Kantarci, 2020). Our previous research has demonstrated that KD exerted a neuro-protective effect on demyelination in mice by improving spatial learning, attenuating social anxiety, reducing astrogliosis and microgliosis, as well as inhibiting demyelination and OLs apoptosis. As KD prominent production of ketones (such as BHB and acetoacetate), BHB has been explored as a neuro-protective agent, which has direct effects on specific transcription factors, inflammation, oxidative stress, mitochondria, epigenetic modifications and the composition of the gut microbiome (Gough et al., 2021). Based on these findings, in the present study, we evaluated the neuroprotective effect of BHB on functional and morphological outcomes following CPZ induced demyelination. Our results indicate that both BHB + CPZ and CPZ + BHB treatments (Zhang et al., 2020) improved the exploratory ability, spatial learning and memory (Liu et al., 2020) suppressed astrogliosis and microgliosis (Klein et al., 2018) resisted demyelination, promoted oligodendrocyte differentiation and supported mature oligodendrocyte survival (Manouchehrinia et al., 2017) induced the release of neurotrophic factors in the brain, such as BDNF and CNTF, and enhanced the production or survival of neuronal cells (Stumpf et al., 2019) decreased the expression of HDAC3, TRPA1, and PARP1 and modulated several signaling pathways, such as ERK1/2, JNK, p38 MAPK, and PI3K/AKT/mTOR.

KD and calorie restriction both can produce ketone bodies, and were shown to be beneficial in some myelinopathies, such as multiple sclerosis and Pelizaeus–Merzbacher disease (Brenton et al., 2019; Stumpf et al., 2019). Our previous research also indicated that high levels of ketone bodies are associated with improved neurological injury outcomes (Liu et al., 2020; Zhang et al., 2020). Here we show that BHB treatment could inhibit demyelination in CPZ-fed mice. In this study, OFT and MWM tests indicated that mice showed significant improvement in exploration, spatial memory and the learning ability in the BHB treated groups as compared to the CPZ group. Moreover, TEM evidences revealed that myelinated axon were observed in the BHB + CPZ and CPZ + BHB mice compared to the CPZ-fed mice. These findings indicate that BHB + CPZ/CPZ + BHB treatment exert a therapeutic effect on demyelination of the CPZ model.

Several studies reported that CPZ induced demyelination is associated with increased density of astrocytes and microglia, excessive astrogliosis and microglial activation that could aggravate neuronal damages (Gerhauser et al., 2012; Hansmann et al., 2019). We previously showed that KD treatment reduced the astrocyte and microglia/macrophage recruitment in the corpus callosum and hippocampus of mouse brain at the 5th week of CPZ induction (Liu et al., 2020; Zhang et al., 2020). The current data of BHB treatment is parallel with our previous research that BHB could also improve the neuronal damages. Furthermore, CPZ triggered a neuro-inflammatory response (such as release of the pro-inflammatory cytokines IL-1β, IL-6 and TNF-α) in the white matter of the central nervous system, which is detrimental to cell survival. Coincidentally, some reports have demonstrated KD or ketone bodies can decrease the pro-inflammatory response and inhibit the demyelination of mice in CPZ and EAE models (Kim et al., 2012; Zhang et al., 2020; Almeida-Suhett et al., 2022). In addition, BHB induction attenuated expression of IL-18, IL-1β and NLRP3 inflammasome in a rodent model of depression (Kajitani et al., 2020). Our results draw similar conclusions to these published reports, BHB treatment can prevent pro-inflammatory cytokine secretion, including IL-1β, IL-6 and TNF-α in CPZ-fed mice. To our knowledge, these results show for the first time that BHB + CPZ or/and CZP + BHB treatment reduced CPZ-induced over activation of astrocyte and microglia, and inhibited pro-inflammatory cytokine secretion.

In this study, we found that BHB treatment inhibited demyelination of CPZ-fed mice. These findings are consistent with previous results that KD has a protective effect on demyelination (Liu et al., 2020; Zhang et al., 2020). It is known that OPCs proliferated and migrated from the subventricular zone and fornix, and then located to the demyelinated lesions to contribute to remyelination. However, the OPC differentiation and maturation was impeded in the pro-inflammatory microenvironment which was mediated by microglia. This may partly explain why an increased number of NG2^+^ OPCs was found in the corpus callosum, but still severe demyelination was observed. Our previous study demonstrated that KD decreased inflammatory response and provided an adaptive benefit for remyelination in CPZ model, which related to the inhibition of HADC3 and NLRP3 expression (Liu et al., 2020). Coincidentally, another report indicated that the down-regulation of HDAC3 expression could promote remyelination and functional neurological recovery in lysophosphatidylcholine induced focal demyelinating lesions model (Ding et al., 2020). Our research showed similar results that both BHB + CPZ and CPZ + BHB treatment diminished inflammatory cytokine release, inhibited the activation of microglia, reduced the density of NG2^+^ OPC cell and decreased the expression of HDAC3, which suggested that BHB treatment could suppress CNS inflammation and inhibited CPZ-induced demyelination might *via* the downregulation of HDAC3 expression. Meanwhile, the higher expression of BDNF and CNTF, and more MCT1^+^ cells were found in the mice of BHB treated group. BDNF and CNTF are known to induce remyelination, and promote neuronal survival. Moreover, MCT1 also palys an important role for neuron survival and appears to be a fundamental property of oligodendroglia. It regulates the lactate export from oligodendroglia and as an important part of local energy supply to axon, while disruption of this transport balance results in axonal dysfunction (Lee et al., 2012). Thus, we concluded that BHB treatment provide a nutritional support for myelin regeneration. Furthermore, Sox2 expresses in OPCs, and promotes OPCs proliferation and differentiation (Savchenko et al., 2019). Another factor, PDGF-Rα, which has a similar function, is also can modulates several aspects of OPCs biology, such as mediating migration and differentiation of OPCs (Calabretta et al., 2018). Herein, we found that BHB treatment increased the expression of Sox2 and the density of PDGF-Ra^+^ Sox2^+^ cells, exerted a neuroprotective function on demyelination by promoting OPCs proliferation and differentiation.

The increase of PARP activation is observed at the later stage of CPZ induction. Over-activation of PARP inducing AIF transports from the mitochondria to the nucleus, which can promote cell apoptosis (Praet et al., 2014). Inhibition of PARP has been shown to ameliorate OLG loss. Veto et al. have observed the therapeutic effect of PARP inhibitor on demyelination disease of the CPZ model by preventing the weight loss and improving the remyelination (Veto et al., 2010). Herein, we revealed that BHB treatment increased the number of APC cells, decreased the expression of PARP and AIF, which suggested that BHB presents a neuroprotective function against oligodendrocyte death. In addition, it has been reported that TRPA1 also plays an key role in the process of CPZ-induced myelin damage (Sághy et al., 2016). TRPA1 expresses on astrocytes and oligodendrocytes in CNS (Sághy et al., 2016; Bölcskei et al., 2018). Bölcskei et al. confirmed that TRPA1 was activated by CPZ induction and promoted cytosolic Ca^2+^ level in oligodendrocytes, thus induced oligodendrocyte cells apoptosis (Bölcskei et al., 2018). Similarly, Sághy et al., 2016 reported that TRPA1 deficiency attenuated CPZ-induced demyelination by reducing the apoptosis of mature oligodendrocytes (Sághy et al., 2016). In this study, less expression of TRPA1 was identified in the BHB supplementation groups, It can be concluded that BHB treatment targets oligodendrocyte apoptosis by reducing the expression of PARP, AIF and TRPA1, thereby inhibiting demyelination in CPZ model.

MAPK pathway (ERK1/2, JNK and p38-MAPK) plays a crucial role in cell survival or stress, such as apoptosis (Veto et al., 2010). Transcription factor C-jun, as a downstream target of JNK and p38-MAPK, is considered to be a pivotal inducer of apoptosis after various CNS insults (Sághy et al., 2016). Since ERK1/2 activation could promote the oligodendrocyte survival (Veto et al., 2010; Sághy et al., 2016), in present study, we observed that BHB induced ERK1/2 activation and exerted a neuro-protective mechanism against cell death of mature OLs. Some studies demonstrated that CPZ-induced apoptosis was mediated by JNK, p38-MAPK and c-Jun activation in the corpus callosum, which can be attenuated by TRPA1 deficiency and PARP inhibition (Veto et al., 2010; Sághy et al., 2016). In this context, the researcher further verified that TRPA1 deficiency enhanced the activation of ERK1/2 in CPZ model. Thus, we evaluated the effects of BHB on MAPK pathways, the down-regulation of p-JNK, p-P38 MAPK and p-cJun, as well as up-regulation of the p-ERK1/2 expression were found in the present study. These data illustrated that BHB treatment has remarkable neuro-protective effect on promoting oligodendrocyte survival and inhibiting mature OLs apoptosis. Although the protective ERK1/2 signaling pathway activated by CPZ was found in this study, this compensatory mechanism seems to be insufficient to prevent cell apoptosis.

Similarly, another interesting signaling pathway, PI3K/AKT signaling, has been confirmed to regulate a wide variety of cellular functions including cell migration and invasion (Wang et al., 2020). mTOR is a direct substrate of the AKT kinase which promotes cell growth, metabolism and survival. In developing oligodendrocytes, mTOR is required for lipid biosynthesis, and myelin growth, which is activated before/during myelination stage (Liu et al., 2017). Liu et al. suggested that PI3K/AKT/mTOR singling regulated OPCs proliferation and differentiation, and promoted CNS remyelination (Liu et al., 2017). Moreover, Akt activation prevented neuronal apoptosis by inhibiting AIF to the nucleus (Kim et al., 2007), and reduced activity of JNK and p38-MAPK (Veto et al., 2010). Consistent with these studies, our research showed that BHB activated PI3K/AKT/mTOR signaling. Thus, we conclude that BHB treatment is contributed to protecting oligodendrocytes against apoptosis and promoting survival of oligodendrocytes partially mediated by decreasing the activation of JNK/p38-MAPK/c-Jun and increasing the activation of PI3K/AKT/mTOR.

In the hippocampus, radial glia (GFAP positive cells), which co-express with Sox2 and Nestin, was considered as stem cells which has the ability to proliferate and differentiate into neurons (Yan et al., 2018). Moreover, it is known that TRPA1 agonists in the hippocampus lead to the depressive-and anxiolytic-like effects, while pharmacological blockage or TRPA1 gene deletion reduce the depression-and anxiolytic-like symptoms. In addition, BDNF is distributed in the hippocampus and participates in neuronal plasticity and facilitating neurons development (Murawska-Ciałowicz et al., 2021). It can also promote the survival and differentiation of myelin-forming cells (Sakita et al., 2016). BDNF/TrkB axis is involved in long-term potentiation and is essential for growth, differentiation and survival of neurons (Sandhya et al., 2013). Targeting TrkB activation is considered to be a strategy for myelin repair in the brain (Nguyen et al., 2019). Interestingly, the present data showed that BHB treatment promoted MBP, BDNF, TrkB and Sox2 expression, as well as enhancing DCX^+^ cells and NeuN^+^ cells production in the hippocampus, which further illustrated BHB favors myelination, promotes cell proliferation and neurogenesis in the hippocampus.

In conclusion, the present study demonstrated the neuro-protective effect of BHB treatment ameliorating behavioral deficits by improving anxiety and alleviating memory impairment in CPZ-fed mice. In addition, BHB treatment could alleviate the demyelination *via* inhibition of the activation of microglia and astrocytes, as well as relieving the neuroinflammatory response, promoting the secretion of neurotrophic factors, preventing oligodendrocyte loss, enhancing OPCs proliferation and differentiation and promoting hippocampal neuron development or persistence in the CPZ-injured mice. Further mechanistic studies showed that BHB exerts neuroprotective effects by down-regulating the expression of TRPA1 and PARP, inhibiting the MAPK pathway (JNK, p38-MAPK and JUN) and activating the ERK1/2 and PI3K/AKT/mTOR signaling pathway. Collectively, our data support BHB as a promising therapeutic agent for future clinical investigations to reduce chronic demyelinating diseases, such as MS.

## Data availability statement

The original contributions presented in the study are included in the article/Supplementary material, further inquiries can be directed to the corresponding authors.

## Ethics statement

All animals received human care in compliance with the guidelines outlined in the National Institutes of Health guide for the care and use of laboratory animals (NIH Publications No. 8023, revised 1978). Moreover, the animal studies in this research were approved by the Animal Ethics Committee of Shandong University (permit number 20191018).

## Author contributions

WS: data curation, formal analysis, software, investigation methodology. MW and ML: conceptualization and software. QL and LL: data curation and formal analysis. QW and RZ: resources, investigation methodology, and project administration. GL and H-CS: supervision and writing-review and editing. NZ: conceptualization, funding acquisition, investigation, writing-original draft, and writing-review and editing. All authors contributed to the article and approved the submitted version.

## Funding

This work was supported by National Natural Science Foundation of China (82001286), Natural Science Foundation of Shandong province (ZR2020QH112 and ZR2021QH022), Open Project of Liaocheng University Animal Husbandry Discipline (319312101–03, 319312101–25 and 319312101–26), Open Project Program of State Key Laboratory of Food Science and Technology, Jiangnan University (SKLF-KF-202112), Youth Innovative Science and Technology Program of Shandong Colleges and University (2021KJ099) and Taishan Scholar Research Foundation (319190201). This work was also technically supported by Shandong Collaborative Innovation Center for Antibody Drugs and Engineering Research Center for Nanomedicine and Drug Delivery Systems.

## Conflict of interest

The authors declare that the research was conducted in the absence of any commercial or financial relationships that could be construed as a potential conflict of interest.

## Publisher’s note

All claims expressed in this article are solely those of the authors and do not necessarily represent those of their affiliated organizations, or those of the publisher, the editors and the reviewers. Any product that may be evaluated in this article, or claim that may be made by its manufacturer, is not guaranteed or endorsed by the publisher.

## Supplementary material

The Supplementary material for this article can be found online at: https://www.frontiersin.org/articles/10.3389/fnagi.2022.1075161/full#supplementary-material
