# Supplementary material for: Effect of β-hydroxybutyrate on behavioral alterations, molecular and morphological changes in CNS of multiple sclerosis mouse model
Source: Front Aging Neurosci. 2022 Dec 1;14:1075161. doi: 10.3389/fnagi.2022.1075161 (PMC9752847; doi:10.3389/fnagi.2022.1075161)
Supplement: Supplementary file 1 [file Data_Sheet_1.doc]

1. **BHB concentration in the mice brain**

The concentration of BHB in the mice brain was detected by mouse β-hydroxybutyric acid ELISA kit (Shanghai Jianglai Industrial Limited By Share Ltd, 226 China), according to manufacturer’s protocol. The levels of BHB in brain of mice significantly increased in the BHB + CP and CPZ + BHB groups, when compared to the control and CPZ groups ( *P<0.05, ^#^P<0.05).


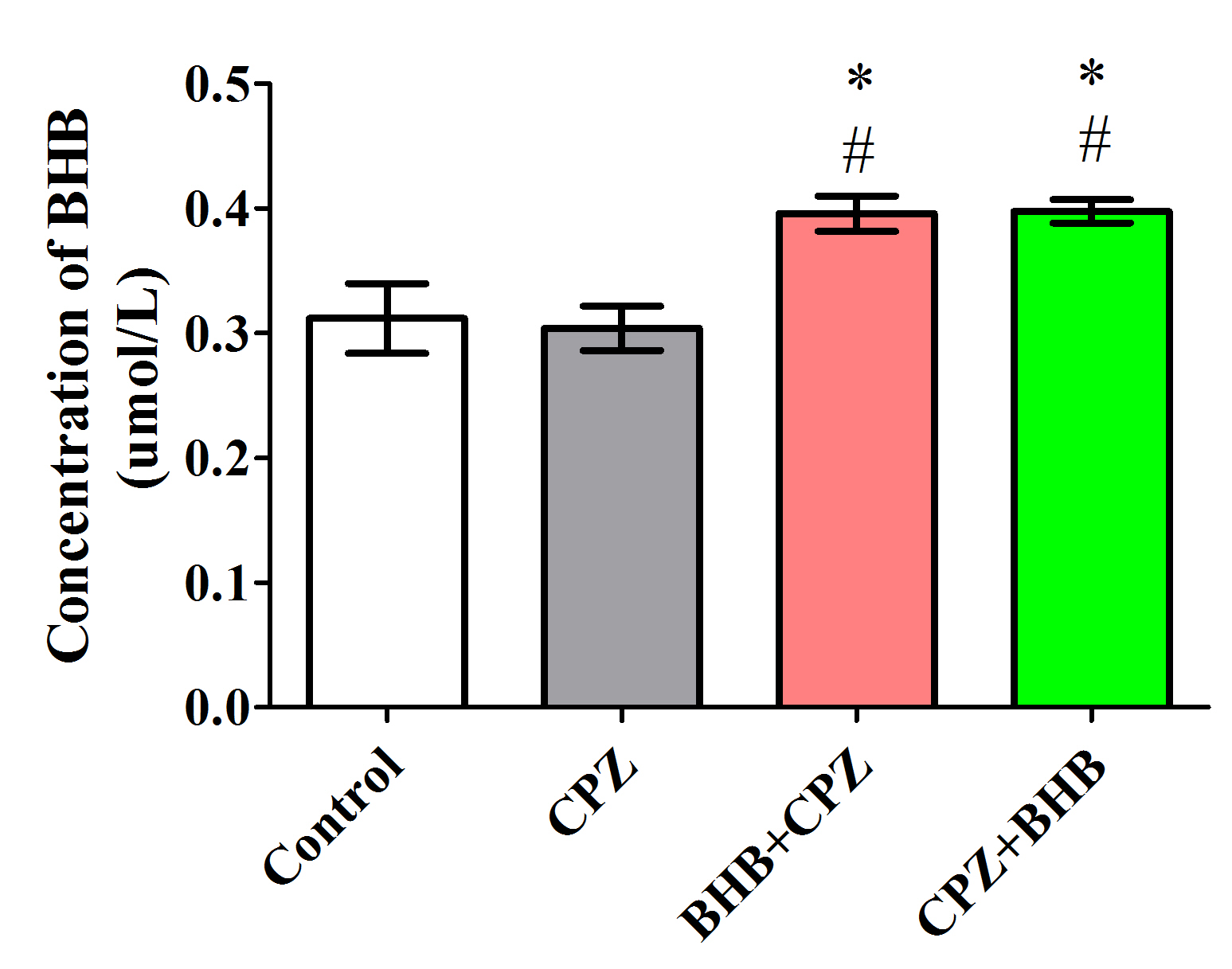


Figure 1. The concentration of BHB was determined in mice with different treatment. N = 3 per experimental group; experiment repeated 2 times. ^*^ P < 0.05, versus the control group; ^#^ P < 0.05 versus the CPZ group.

1. **Immunohistochemical staining of DCX in the mouse dentate gyrus subgranular zone.**

Immunohistochemical detection of DCX^+^ (Figure 2) revealed that more DCX^+^ cells in the BHB treated groups than those in the CPZ group, indicating that BHB affect neurogenesis.

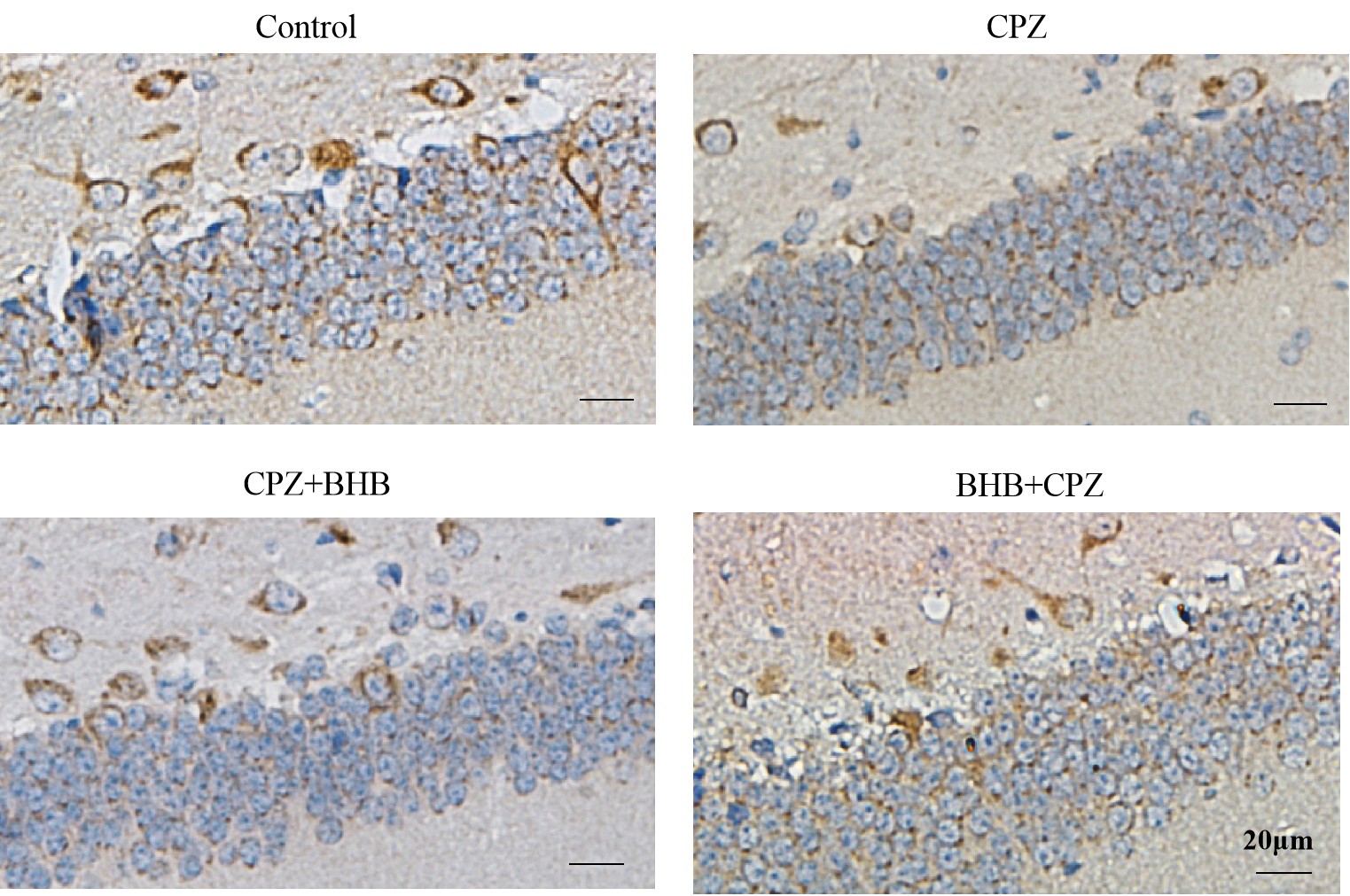


Figure 2. Images of the granule cell layer of the dentate gyrus in mice stained for DCX^+^.
